# Predictors of inadequate and excessive gestational weight gain in women: a retrospective longitudinal observational study

**DOI:** 10.1136/bmjopen-2024-087589

**Published:** 2025-03-22

**Authors:** Sanjeeva Ranasinha, Joanne Enticott, Cheryce Harrison, Helena J Teede

**Affiliations:** 1 Monash Centre for Health Research and Implementation, Monash University, Melbourne, Victoria, Australia; 2 Faculty of Medicine, Nursing and Health Sciences, Monash University, Clayton, Victoria, Australia; 3 Alfred Health, Melbourne, Victoria, Australia; 4 Monash Health, Melbourne, Victoria, Australia

**Keywords:** EPIDEMIOLOGY, Hospitals, Public health, Paediatric intensive & critical care, GENERAL MEDICINE (see Internal Medicine), Health policy

## Abstract

**Abstract:**

**Importance:**

Monitoring and predicting optimal gestational weight gain (GWG) is important for maternal and child health. However, with recommendations based on total pregnancy GWG, available tools for real-time use in pregnancy care are lacking. These tools are prioritised by the WHO to enable healthcare providers to identify, monitor and target lifestyle interventions for those at high risk of suboptimal GWG and subsequent adverse health outcomes for mothers and babies.

**Objective:**

This study aims to identify risk factors associated with GWG and to use these to develop an antenatal risk prediction tool for use during pregnancy to guide healthcare providers and women on optimal GWG, based on early pregnancy weight gain data.

**Design:**

Routine health data from the Australian Monash Health Network birthing outcome system were used to analyse GWG in women of different body mass index (BMI) categories. Using data from 10 to 15, 15–20 and 15–25 weeks of pregnancy, we predicted the probability of women gaining inadequate or excessive total GWG by term. We used multinomial logistic regression to investigate associations between US National Academy of Medicine (NAM) classifications (inadequate, sufficient and excessive GWG) and BMI, age, country of birth (COB) by region, parity, socioeconomic status and visit frequency.

**Setting:**

We used individual patient data routinely collected during care from one of the largest antenatal health networks in Australia.

**Participants:**

The study included 17 397 women from 149 countries (based on the COB) of diverse socioeconomic backgrounds, with pregnancies between 2017 and 2021.

**Exposure:**

Gestational weight gain.

**Results:**

Overall, 31.5% gained below, 35.7% within and 32.8% above NAM GWG recommendations. Risk factors for excess GWG were higher BMI and maternal COB by region. Compared with the healthy BMI group, the overweight group has a 4.05 times higher adjusted relative risk of excess GWG (95% CI 3.37 to 4.80), and the obese group had a relative risk of 6.64 (95% CI 5.27 to 8.37). The risk prediction tool receiver operating characteristic curve was 0.81 for the 15–25 week, 0.80 for the 15–20 week and 0.69 for the 10–15 week GWG groups, with excellent performance in both discrimination and reliability.

**Conclusions and relevance:**

From a large population of women from diverse socioeconomic backgrounds, we have identified risk factors for suboptimal GWG and developed and internally validated a risk prediction tool for attainment of recommended GWG from early pregnancy, with high performance. This tool is designed to enable clinicians to prospectively predict attainment of NAM GWG recommendations to guide risk stratification, monitoring and appropriate intervention for those at risk of suboptimal GWG.

**STRENGTHS AND LIMITATIONS OF THIS STUDY**

- This study has several strengths including a diverse cohort across multiple large-scale maternity hospitals and detailed capture of gestational weight gain (GWG) during pregnancy. As a general population-based cohort study, it provides a good representation of different socioeconomic groups and a fair representation of the prevalence of inadequate and excess GWG. It was based on routinely collected data, and the risk prediction tool had good performance with excellent reliability.
- Limitations include that categorising ancestry is challenging, with no agreed global approach. Although clinical measurements of height and weight are considered the gold standard, self-reported measurements are a reasonable proxy and are used widely in the literature.^29 30^
- Classifying ethnicity by the country of birth may have introduced some inaccuracies; therefore, we aim to improve on this by exploring self-identified ethnicity in the future.

## Introduction

 The National Academy of Medicine (NAM) defines gestational weight gain (GWG) as the change in weight from prepregnancy (or first trimester) to weight at term. GWG is increasing worldwide, and excess GWG is prevalent, leading to pregnancy risks.^1^ The NAM recommendations for healthy GWG were based on data from the USA, with weight gain outside these recommendations significantly impacting maternal and child health.^1 2^ Our systematic reviews of 1.3 million women found that, when applying NAM recommendations internationally, 47% exceeded recommendations and 23% had insufficient GWG.^1^ Excess GWG is associated with increased gestational diabetes and hypertension, preterm birth, large for gestational weight (LGA) infant, caesarean delivery, postpartum weight retention and childhood obesity, whereas insufficient GWG is associated with increased preterm birth and small for gestational age infants.^13^,^5^ The 2009 NAM guideline recommendations are stratified by a woman’s baseline body mass index (BMI) categories aligned to the WHO; underweight (BMI ≤ 18.5 kg/m^2^, GWG of 12.5–18 kg), healthy weight (18.5–24.9, GWG of 11.5–16 kg), overweight (25.0–29.9, GWG of 7–11.5 kg) and obese (≥30.0, GWG of 5–9 kg) as classified by the NAM.^2 6^

Antenatal lifestyle interventions improve attainment of recommended GWG and unequivocally improve the health of women and infants, with evidence of efficacy, cost-effectiveness and cost savings.^7^ The US Prevention Taskforce now recommends antenatal lifestyle intervention to optimise GWG.^8^ In this context, monitoring and intervention to optimise GWG during pregnancy is key for the majority of women with GWG outside recommendations. Yet studies on optimal GWG are largely retrospective and focused on total GWG. Tools for real-time GWG monitoring are lacking but are vital for personalised antenatal prevention and care. GWG trajectories vary throughout pregnancy, and first trimester GWG may better predict certain infant outcomes (increased birth weight), child (overweight/obesity and cardiometabolic risk) and mother (gestational diabetes and weight retention). Prediction of GWG outside recommendations from early pregnancy offers the opportunity to identify those at risk, monitor, and intervene with targeted lifestyle intervention aligned to WHO priorities for the development of international reference standards for GWG.

We aimed to address this gap and WHO priority by first exploring risk factors associated with suboptimal NAM-recommended GWG associated with GWG between 15 and 25 weeks. Second, we aimed to develop a total GWG risk prediction tool from early GWG data at 10–15, 15–20 and 15–25 weeks to identify those at risk, monitor and guide targeted intervention during pregnancy to improve maternal and neonatal outcomes.

## Methods

### Study design and setting

The first aim is addressed through a retrospective longitudinal observational study, while the second aim involved developing an algorithm for risk prediction. We used individual patient data routinely collected during care, from the largest antenatal health network in Australia. This network serves a diverse population across countries of birth and socioeconomic backgrounds within a universal government-funded healthcare system.

Results were reported according to the RECORD statement for routinely collected health data, Strengthening the Reporting of Observational Studies in Epidemiology (STROBE) guidelines for risk factor analysis^9^ and Transparent Reporting of a multivariable prediction model for Individual Prognosis or Diagnosis (TRIPOD) guidelines for risk prediction algorithm development.^10^

### Data source and sample

The Birthing Outcomes System (BOS) is an electronic database of routinely collected data, used to gather demographic information, such as age, country of birth (COB) by region, parity, socioeconomic status (SES), obstetric history, anthropometric and blood measurements at routine visits, and pregnancy outcome data. All singleton pregnancies from 2017 to June 2021 were used across the full pregnancy and birth. Of the 17 397 pregnancies, after missing values, we analysed 11 639 for the 15–25 week period. For the second aim, we generated three datasets: 15–25, 15–20 and 10–15 weeks GWG to predict NAM GWG below, within or above recommendations.

### Variables and measurements

GWG was from the BOS hospitals’ computerised records at each visit. Maternal prepregnancy BMI was categorised as underweight (< 18.5 kg/m^2^), healthy weight (18.5–24.9 kg/m^2^), overweight (25.0–29.9 kg/m^2^) or obese (≥30 kg/m^2^) by WHO criteria. COB BMI categories were applied for Chinese and Korean-born women, aligned to WHO BMI categories, previously validated for GWG recommendations;^11^ China and Korea: both underweight BMI < 18.5 kg/m^2^, normal weight 18.5–23.9 and 18.5–22.9 kg/m^2^, overweight 24–28 and 23–25 kg/m^2^, and obese ≥28 and ≥ 25 kg/m^2^, respectively.^11^ GWG was calculated as the difference between weight at a certain gestational age and prepregnancy weight. Region was determined by COB, categorised into 12 regions by Australian Bureau of Statistics categories.^12^

### Ethics statement

Participant data were deidentified, and consent was not directly obtained. The project was approved by the Monash Health Human Research Ethics Committee (project no. RES-21–0000183 L).

### Statistical analysis

GWG was determined by subtracting self-reported prepregnancy weight from the antenatal visit measured weight. At the final visit (≥37 weeks), women were classified into GWG below, within or above NAM-recommended GWG by the four BMI categories. The cohort characteristics are presented as means (SD) for continuous variables and frequencies (%) for categorical variables. Comparisons between groups used analysis for variance (ANOVA) and χ^2^ tests.

A multivariable multinomial logistic regression model was created to investigate relationships between risk factors and GWG outside NAM guidelines with recommended gain as the reference category. In the multivariable analysis, variables with a p value <0.1 on bivariable analysis were included. Multivariable models included the covariates of age, region, parity, BMI, GWG between 10 and 15, 15 and 20, and 15 and 25 weeks, number of maternal visits, and SES.

Associations were reported using relative risk and 95% CIs. GWG between 15 and 25 weeks was used in the primary analysis to examine the association between NAM recommendations and selected covariates with 10–15 and 15–20 weeks as secondary analyses.

For the second aim, the NAM classification algorithm involved three steps. The weight during the specified weeks was used to predict GWG by NAM classification at ≥37 weeks. If the GWG was suboptimal, it was further validated using NAM classifications at ≥39 weeks. This process was repeated for women visiting between 15–20 and/or 15–25 weeks of pregnancy. The Kappa statistic was employed to test the concordance between these steps. Internal validation was performed to predict outcomes in the validation set by dividing the data into separate training and validation datasets (60% (n=6983)/40% (n=4566)). The model’s ability to classify NAM GWG recommendations was evaluated using computed probabilities and multiclass receiver operating characteristic curve (ROC). Multiclass ROC evaluates risk tool classification accuracy by comparing bootstrapped cases (100 runs) with left-out cases, leaving multinomial class labels intact (alternative/model accuracy), with the procedure repeated with labels randomly assigned (null accuracy). With these runs, smoothed probability distributions were computed using kernel density estimation, and the false positive rate, true positive rate and area under the curve (AUC) were computed.^13^ Statistical analyses were conducted using R and Stata V.14.

## Patient and public involvement

Patients and/or the public were not involved in this study as this is an observational study.

## Results

### Aim 1: Examine the relationships between NAM GWG classifications inadequate, sufficient and excessive GWG with BMI, COB by region, age, parity, SES and number of visits

A primary analysis of 17 397 women revealed that 31.5% of women gained insufficient weight, 35.7% sufficient weight and 32.8% gained excess weight according to NAM GWG recommendations. Mean age was 30.1±5.0 years and mean BMI 25.8±5.5 kg/m^2^. Analysis by region showed 6528 (37.5%) were Caucasian, 3919 (22.5%) South-Central Asian and 1681 (9.7%) Central Asian by maternal COB, and for 7612 (43.7 %) were nulliparous, 9252 (53.2 %) had 1–3 children and 533 (3.1 %) had more. Analysis of SES revealed 4729 (27.2%) classified in the highest SES group, 6554 (37.7%) in the middle and 3648 (21.0%) in the lowest tertile (table 1).

**Table 1:** NAM analysis on the complete data; characteristics of the participating pregnancy cohorts

Characteristic	GWG below NAM (n=5482)	GWG within NAM (n=6213)	GWG exceeds NAM (n=5702)	P value
Age (years)				
<25	718 (28.8)	766 (31.8)	928 (38.5)	<0.001*
25–29	1556 (28.7)	1950 (35.9)	1925 (35.4)	
30–34	2027 (32.2)	2339 (37.1)	1932 (30.7)	
≥35	1181 (36.3)	1158 (35.6)	917 (28.2)	
BMI categories				
Underweight (<18.5)	253 (48.0)	213 (40.4)	61 (11.6)	<0.001*
Normal weight (18.5–25.9)	3507 (40.5)	3349 (38.7)	1799 (20.8)	
Overweight (26.0–29.9)	955 (19.1)	1742 (34.8)	2316 (46.2)	
Obese (≥30)	767 (24.0)	909 (28.4)	1526 (47.7)	
Offspring birth weight (g) (mean, SD)	3272 (± 439)	3408 (± 429)	3544 (± 464)	<0.001
Parity (%)				<0.001*
0	2082 (27.4)	2667 (35.0)	2863 (37.6)	
1–3	3174 (34.3)	3359 (36.3)	2719 (29.4)	
>3	226 (42.4)	187 (35.1)	120 (22.5)	
Mothers’ weight (mean, SD)	64.9 (± 16.8)	66.0 (±14.9)	73.1 (±16.3)	<0.001
Country of birth (%)				<0.001*
Australian/European†	1697 (26.0)	2126 (32.6)	2705 (41.4)	
Polynesian	62 (24.3)	71 (27.8)	122 (47.8)	
South-East Asian	685 (42.2)	661 (40.7)	277 (17.1)	
Maritime SE Asian	286 (37.1)	282 (36.6)	202 (26.2)	
NE Asian	281 (32.9)	351 (41.2)	221 (25.9)	
South Central Asian	1330 (33.9)	1474 (37.6)	1115 (28.5)	
African	267 (45.0)	206 (34.7)	121 (20.4)	
Indigenous/Torres Strait Isle	38 (35.9)	30 (28.3)	38 (35.9)	
South Central America	31 (34.4)	31 (34.4)	28 (31.1)	
Central Asian	555 (33.0)	639 (38.0)	487 (28.0)	
Southeastern Europe	147 (24.5)	207 (34.6)	245 (40.9)	
North Africa and the Middle East	54 (23.6)	87 (38.0)	88 (38.4)	
Other	49 (32.7)	48 (32.0)	53 (35.3)	
Socioeconomic deprivation quintile (1=high, 5=low)				<0.001*
1	1571 (33.2)	1720 (36.4)	1438 (30.4)	
2	343 (33.3)	369 (35.8)	318 (30.9)	
3	2035 (31.1)	2312 (35.3)	2207 (33.7)	
4	420 (29.3)	545 (38.0)	471 (32.8)	
5	1113 (30.5)	1267 (34.7)	1268 (34.8)	
No. of visits for the duration of pregnancy				<0.001*
1–3	1512 (40.2)	1202 (32.0)	1043 (27.8)	
4–7	775 (30.9)	916 (36.5)	819 (32.6)	
≥ 8	3195 (28.7)	4095 (36.8)	3840 (34.5)	
Maximum GWG (kg) 10–15 weeks n (mean; 95% CI)	8.5 (8.0 to 9.0)	12.4 (1.9 to 12.8)	17.0 (16.4 to 17.6)	<0.001
Maximum GWG (kg) 15–20 weeks n (mean; 95% CI)	7.9 (7.4 to 8.4)	11.7 (11.8 to 12.1)	15.9 (15.3 to 16.6)	<0.001
Maximum GWG (kg) 15–25 weeks n (mean; 95% CI)	6.9 (6.4 to 7.3)	10.4 (9.9 to 10.9)	15.7 (14.8 to 16.6)	<0.001

Analysis of variance was used to determine the p value.

Other include Bahamas, Brunei, Darussalam, Dominican Republic, East Timor, Equatorial Guinea, Israel, Lesotho, Malta, Not Stated, South Africa.

* Test was used to determine the p value.

† Australia, Belgium, Canada, Denmark, England, France, Germany, Ireland, Italy, Netherlands, New Zealand, Northern Ireland, Norway, Portugal, Scotland, Sweden, Switzerland, UK, Channel Islands & Isle, USA, Wales.

§ Only 217 women, or 1.2%, were under 20 years.

BMIbody mass indexGWGgestational weight gainNAMNational Academy of Medicine

Characteristics for the 15–25 week cohort (n=11 639), included 47.6% with a high BMI and 3% underweight, while 33.3% gained excess GWG and 32.2% insufficient GWG, with 65.5% outside NAM guidelines (online supplemental table S1).

#### Multivariable regression analysis

**Figure 1:**
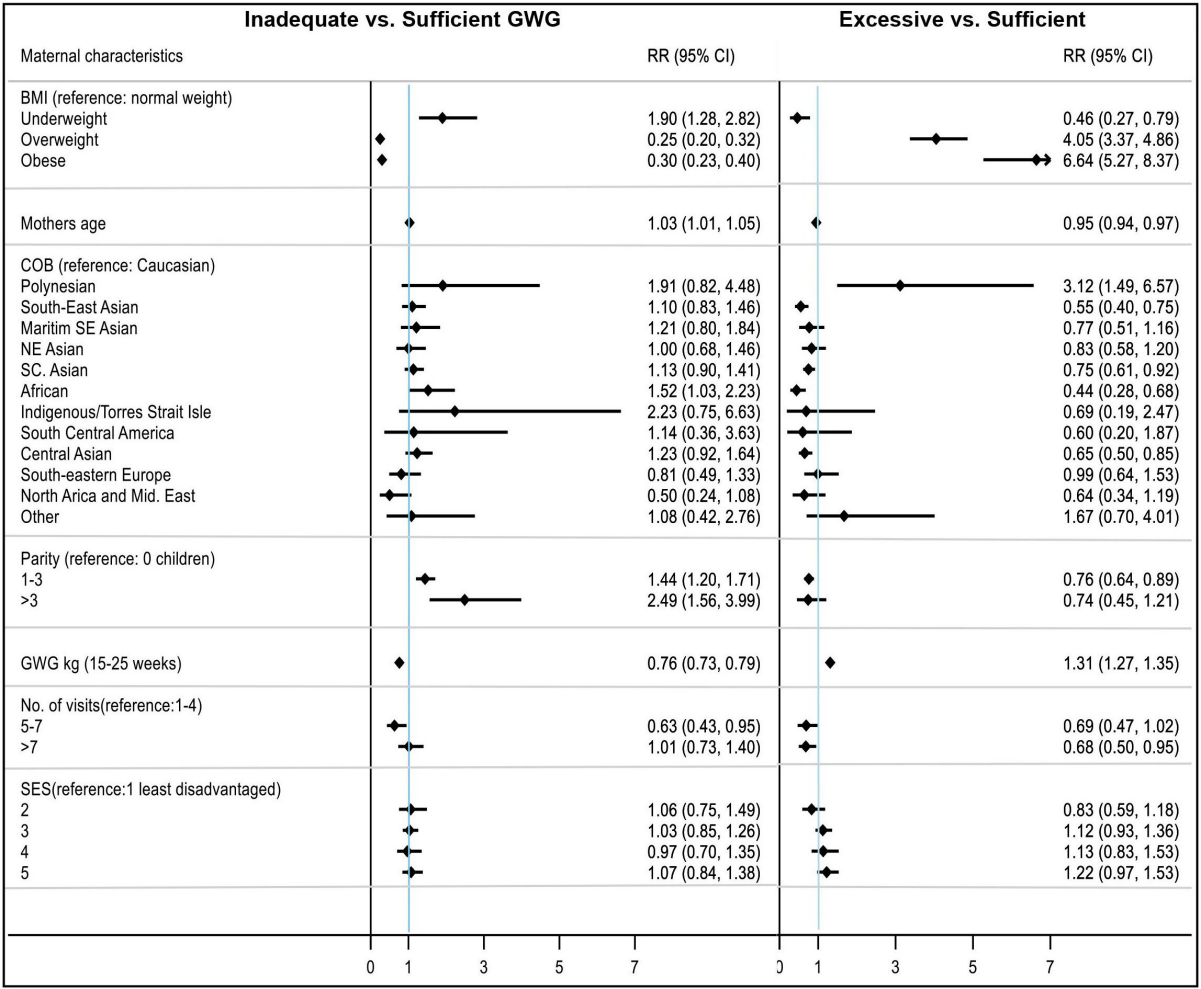
Excessive versus sufficient GWG maternal characteristics associated with NAM classifications for gestational age 15–25 weeks. COB, country of birth; GWG, gestational weight gain; NAM, National Academy of Medicine; RR, relaive risk.

Figure 1 summarises multivariable relationships between total GWG NAM classifications and GWG at 15–25 weeks, BMI, COB by region, age, parity, SES and number of visits, all being statistically significant on bivariable analysis. The 15–20 and 10–15 weeks GWG results are in online supplemental figures S1 and S2.

#### Inadequate GWG

Inadequate GWG was associated with maternal underweight, with an adjusted relative risk ratio (aRRR) of 1.90 (95% CI 1.28 to 2.82) versus healthy weight women. Indigenous women had an increased aRRR of 2.23 (95% CI 0.75 to 6.63) and those of African origin, an aRRR of 1.52 (95% CI 1.03 to 2.23) of gaining below NAM recommendations, compared with Caucasians. Multiparous women with 1–3 children had an aRRR of 1.44 (95% CI 1.20 to 1.71) and women with >3 children produced an aRRR of 2.49 (95% CI 1.56 to 3.99) versus nulliparous women.

#### Excess GWG

Women in overweight and obese groups had an aRRR of 4.05 (95% CI 3.37 to 4.80) and 6.64 (95% CI 5.27 to 8.37), compared with healthy weight women. Women of Polynesian origin had an aRRR of 3.12 (95% CI 1.49 to 6.57). Each 1 kg increase in GWG at 15–25 weeks resulted in an increased aRRR of 1.31 (95% CI 1.27 to 1.35) of gaining excessive GWG. Multiparous women had a reduced risk of excess GWG with one to three children (aRRR 0.76 (95% CI 0.64 to 0.89)) and more than three children (aRRR 0.74 (95% CI 0.45 to 1.21, ns)). Higher antenatal visits were positively associated with less excess GWG, with 5–7 visits having an aRRR of 0.69 (95% CI 0.47 to 1.02, ns), compared with 1–4 visits. SES was not significantly related to suboptimal NAM GWG recommendations (figure 1).

#### Defining relationships between GWG and other factors between 15 and 25 weeks gestation

**Figure 2:**
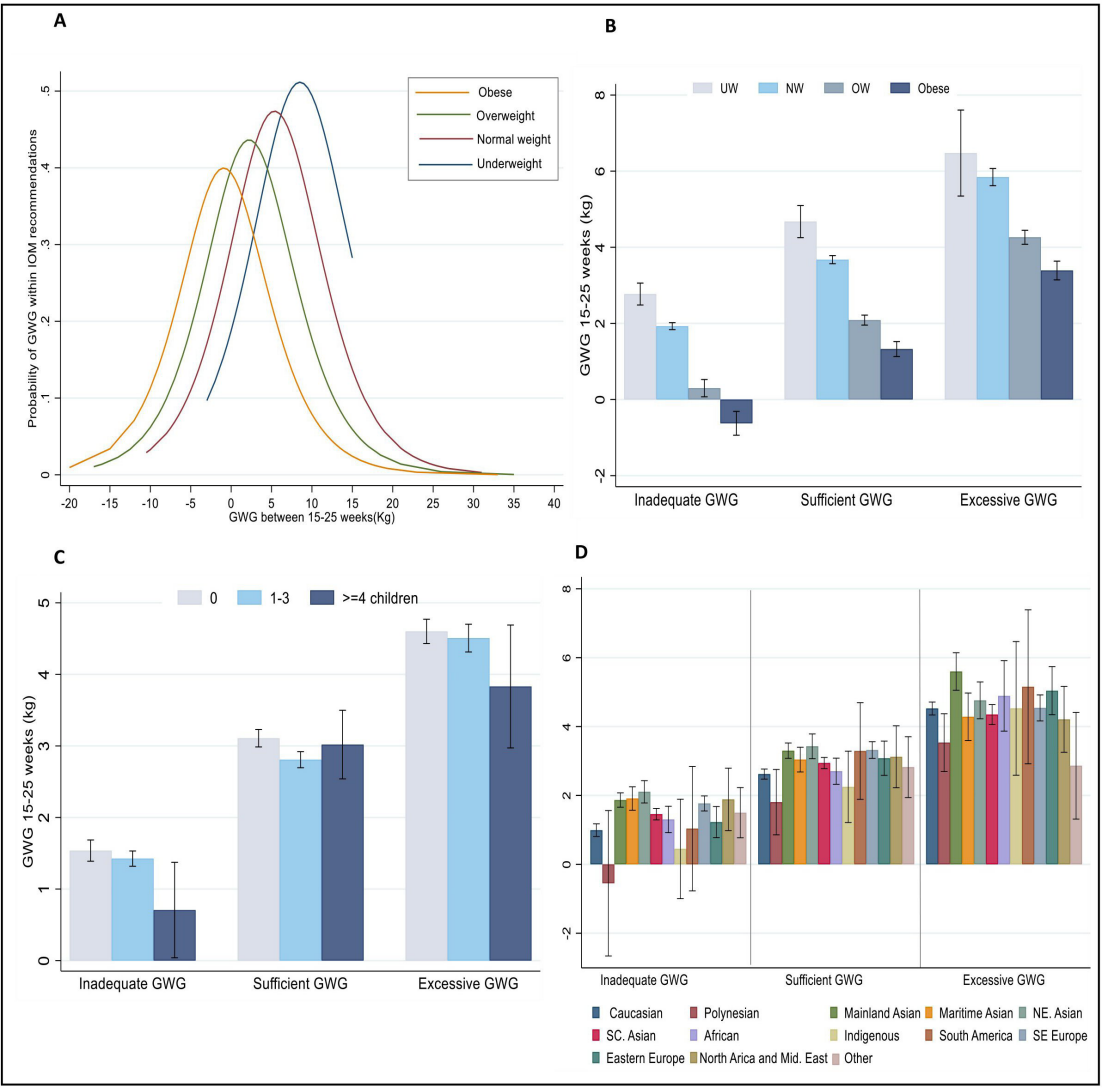
(A) Probability of being within NAM recommendations by BMI categories, (B) GWG at 15–25 weeks against explanatory variables BMI, (C) parity and (D) proportion of women by country of birth and NAM guidelines. BMI, body mass index; GWG, gestational weight gain; NAM, National Academy of Medicine.

Analysis of GWG between 15 and 25 weeks revealed that different BMI categories had variable probabilities of achieving GWG recommendations by term (figure 2A, online supplemental table S2). NAM GWG classifications by BMI groups are in figure 2B at 15–25 week gestational age.

Nulliparous women gained 3.1 kg (95% CI 2.9 to 3.2), women with one to three children gained 2.8 kg (95% CI 2.7 to 2.9) and women with more than three children gained 3.0 kg (95% CI 2.5 to 3.4), all within NAM recommendations (figure 2C). GWG at 15–25 weeks by COB by region showed that GWG at 15–25 weeks varied by maternal COB (figure 2D).

#### Proportionality of BMI by COB by region, age and parity

**Figure 3:**
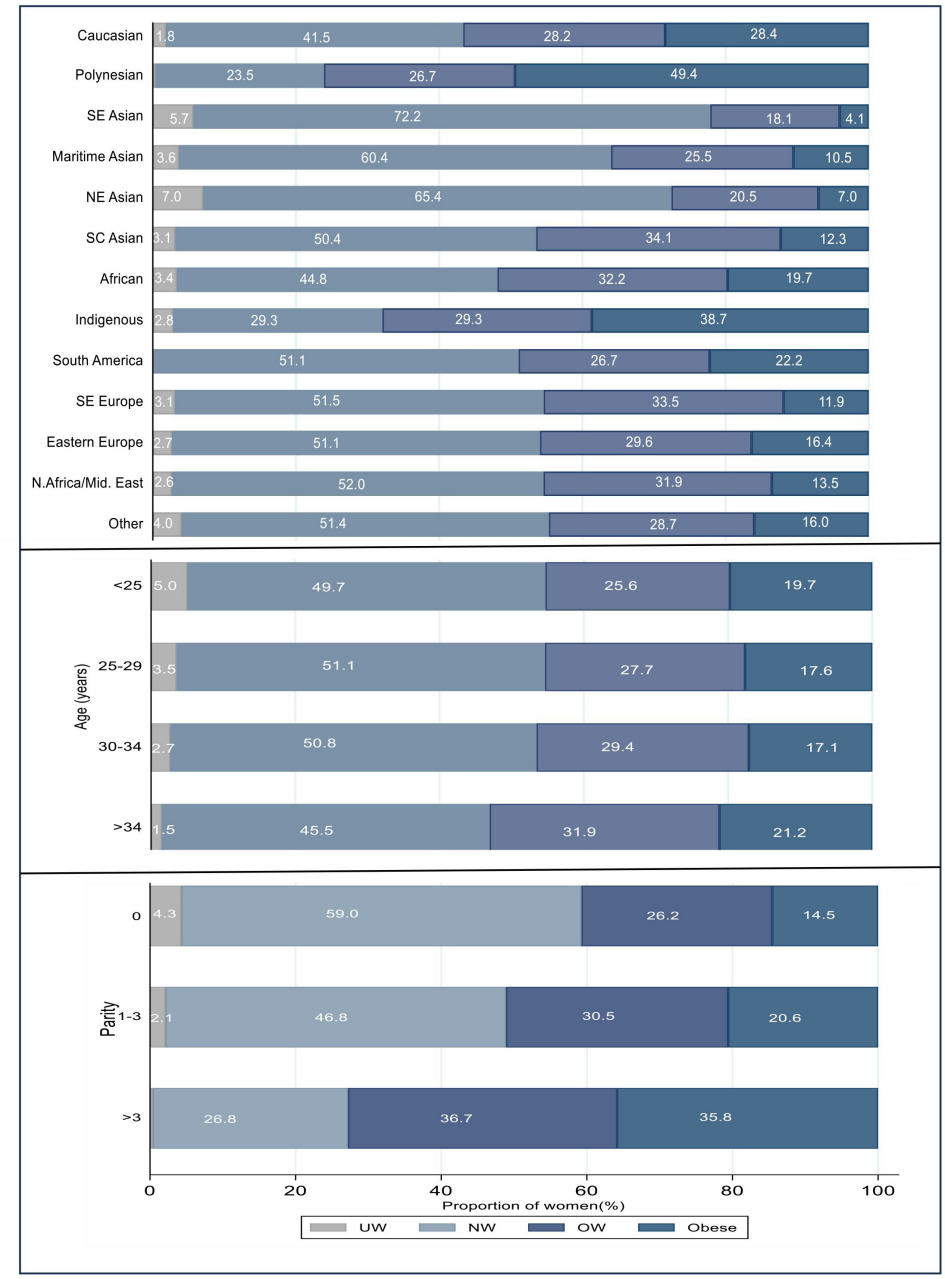
Proportion of women by body mass index for country of birth, age and parity.

The analysis of the distribution of BMI by region, parity and maternal age is shown in figure 3, with COB with high BMI also had higher risk of excess GWG. Polynesians had a 49.4% obesity rate and Indigenous/Torres Strait islanders (38%) compared with Caucasians at 28.4%. Southeast Asian, Northeast Asian and Maritime Asian women had lower obesity prevalence (4.1%, 7.0% and 10.5%).

Analysis by age and BMI did not reveal clinically significant differences (age <25, mean BMI=25.7, SD (6.0); age 25–29, mean BMI=25.6, SD (5.2); age 30–34, mean BMI=25.6, SD (5.2); age ≥34, mean BMI=26.3, SD (5.3)). BMI distribution by parity revealed increasing proportion in higher BMI groups with higher parity (p<0.001).

### Aim 2 Risk prediction algorithm

#### Performance

For an individual, her final GWG can be predicted using her weight gained earlier in the pregnancy. We examined the predictive performance using three times in early pregnancy. Classification accuracy was evaluated with multiclass ROC analyses,^9^ with GWG at 15–25 AUC being 0.81 (online supplemental figure S3), at 15–20 weeks 0.80 (online supplemental figure S4) and at 10–15 weeks 0.69 (online supplemental figure S5). Internal validation for the 15–25 week cohort produced correct classification of 88% for the 15–25 week cohort, 80.3% for the 15–20 week cohort and 61.4% for the 10–15 week cohort, on respective testing data sets. Concordance between 10–15 weeks GWG and 15–20 weeks GWG predictions was 75% (online supplemental table S3). Concordance between the ≥37 week NAM classification for 10–15 weeks GWG and the >39 week NAM classification was 96%, indicating excellent agreement between the NAM classification cohorts (online supplemental table S4).

## Discussion

This study used routine antenatal data from a diverse population of women born across 149 countries and first identified risk factors for suboptimal GWG with BMI, COB and parity as key factors. Second, a robust risk prediction tool was developed for suboptimal GWG at term, performing well with an ROC of 0.81 for the 15–25 week group, 0.80 for the 15–20 week group and 0.69 for the 10–15 week group. Internal validation showed 88% correct classification for the 15–25 week, 80.3% for the 15–20 cohort and 61.4% for the 10–15 cohort, indicating good discrimination.

Excess GWG during pregnancy is independently associated with adverse outcomes for both the mother and the child, leading to long-term non-communicable diseases in women and epigenetic consequences across generations.^14^ The revised NAM guidelines for total pregnancy GWG do not enable healthcare providers to identify those with excess GWG during pregnancy to guide prevention practices and optimise health outcomes. Furthermore, GWG follows different trajectories across pregnancy, and early GWG may better predict some infant,^15^ child^16 17^ and maternal outcomes^18 19^ compared with GWG later in pregnancy. Our study confirmed high BMI is a key risk factor for excess GWG, while low BMI was significantly associated with insufficient GWG. GWG can differ substantially by maternal COB,^20^,^22^ with this variable emerging as an important independent predictor of suboptimal GWG. Nulliparity was also a risk factor for higher GWG, as previously shown.

GWG is a modifiable risk factor, and our original research and systematic review and meta-analysis found that antenatal structured diet and physical activity-based lifestyle interventions were associated with reduced GWG and with maternal and neonatal health benefits.^14 23^

The socioeconomic status had minimal impact on consumer visits (online supplemental figure S6).

The US Preventive Services Task Force has now recommended implementation of antenatal lifestyle interventions to limit excess GWG, with structured diet and physical activity interventions shown to be the most effective.^24 25^ However, to identify those at risk, stratify care, monitor progress and target lifestyle interventions, total GWG is unhelpful and strategies for application in early pregnancy are vital. In this study, we have advanced knowledge to address this gap, applied optimal methods and created and validated a risk prediction tool with good performance. The prediction tool involved a robust three-stage gated sequential approach to optimise the accuracy and reliability of the prediction tool. This algorithm had an inter-rater agreement between GWG models at 10–15 weeks and 15–20 weeks of 75%, indicating substantial agreement.^26^ The concordance of NAM GWG classification derived from the models between the ≥37 GWG cohort and the >39 week cohort yielded very high agreement between the two cohorts (95.5%) indicating excellent model reliability.

**Figure 4:**
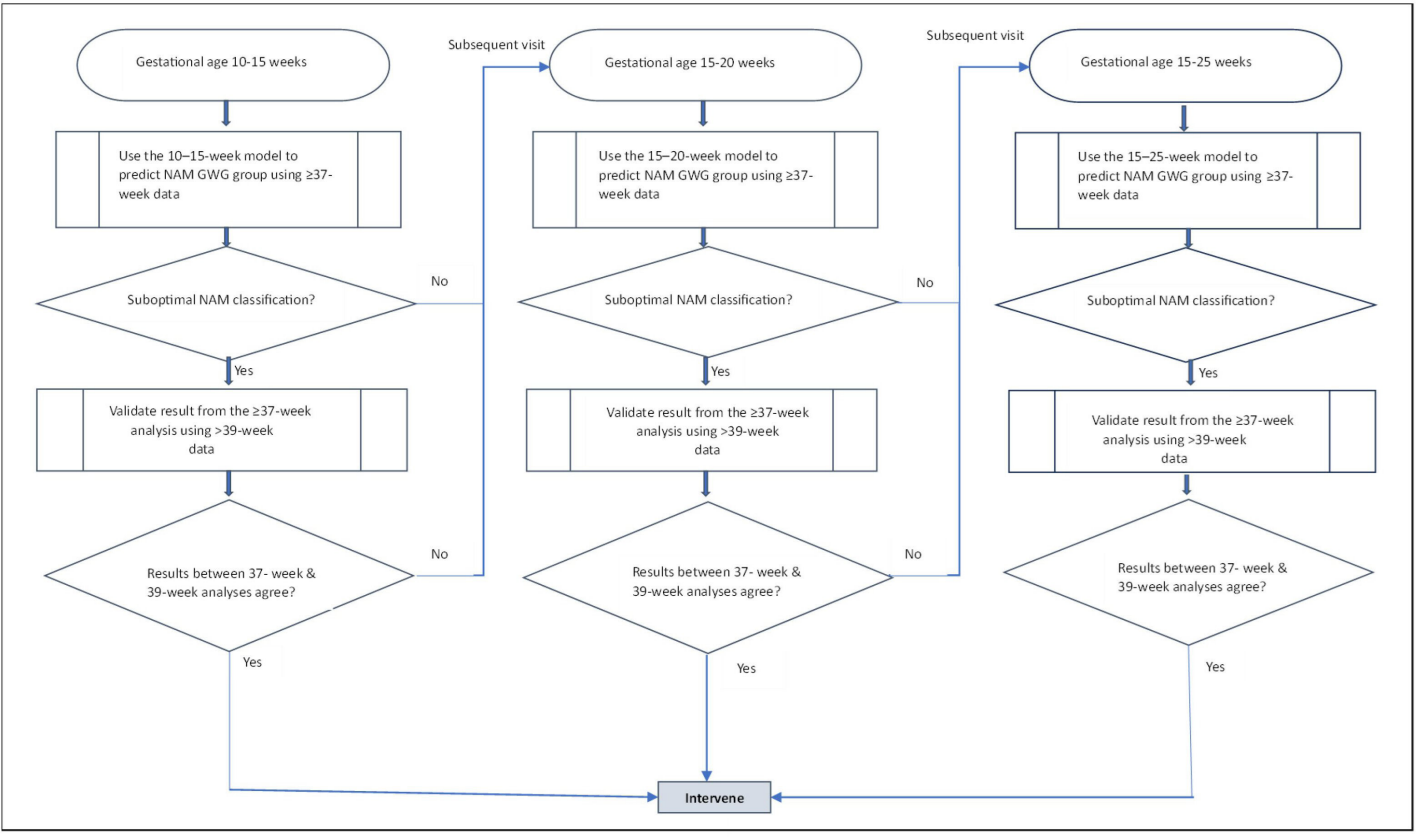
Flow diagram of the proposed risk prediction model. GWG, gestational weight gain; NAM, National Academy of Medicine recommended GWG at term (insufficient, adequate, excess GWG); suboptimal NAM classification, insufficient or excessive GWG.

Several studies have investigated factors related to excessive or insufficient GWG.^27 28^ However, these were exploratory studies with limited generalisability. This study employs easily accessible, routinely collected covariates that were used in the risk prediction tool. The prediction tool is a robust three-stage algorithm that uses a gated sequential approach. In the first stage, we use the GWG at 10–15 weeks to derive a classification based on the ≥37 week NAM classification. This result is validated using the NAM classifications at >39 weeks. This process is repeated for women who visit between 15–20 and 15–25 weeks into pregnancy. This ensures the accuracy and reliability of the prediction tool. This algorithm is illustrated in figure 4. Previous studies have not adopted the risk prediction approach based on early GWG and covariates in the personalised risk prediction tool for total GWG. The present study offers a novel and relatively simple strategy reliant on clinically collected variables in routine care.

### Strengths and limitations

This study has several strengths including a diverse cohort across multiple large-scale maternity hospitals and detailed capture of GWG during pregnancy. As a general population-based cohort study, it provides a good representation of different socioeconomic groups and a fair representation of the prevalence of inadequate and excess GWG. It was based on routinely collected data, and the risk prediction tool had good performance with excellent reliability. Limitations include that categorising ancestry is challenging, with no agreed global approach. Classifying ethnicity by the COB may have introduced some inaccuracies; therefore, we aim to improve on this by exploring self-identified ethnicity in the future. Although clinical measurements of height and weight are considered the gold standard, self-reported measurements are a reasonable proxy and are used widely in the literature.^29 30^

### Conclusion

We have evaluated a large population-based and diverse cohort across COB and SES and have identified characteristics associated with suboptimal GWG and developed and internally validated a risk prediction tool for suboptimal GWG at birth, using early pregnancy GWG and other relevant covariates. This generalisable knowledge can identify women in early pregnancy at high risk of suboptimal GWG, guiding clinicians in risk stratification, monitoring and targeted lifestyle interventions to prevent suboptimal GWG, improve health outcomes and reduce health and cost burdens.

### Key points

Questions: What are the key features associated with suboptimal gestational weight gain (GWG) during pregnancy, and what is the most accurate algorithm to predict those at risk of GWG outside recommendations?

Findings: In this cohort study of 17 397 women from 149 countries of diverse socioeconomic backgrounds, the primary risk factors associated with excess GWG were higher body mass index (BMI) and country of birth (COB). The risk prediction tool, including these parameters, had excellent performance in both discrimination and reliability.

Meaning: These findings generated from a diverse population accurately identify those at risk of suboptimal GWG and subsequent adverse pregnancy outcomes during pregnancy. This can guide risk stratification, monitoring, and targeted intervention for those at risk of suboptimal GWG.

## supplementary material

10.1136/bmjopen-2024-087589 online supplemental file 1

## Data Availability

Data may be obtained from a third party and are not publicly available.
