# Supplementary material for: Predictors of inadequate and excessive gestational weight gain in women: a retrospective longitudinal observational study
Source: BMJ Open. 2025 Mar 22;15(3):e087589. doi: 10.1136/bmjopen-2024-087589 (PMC11931920; doi:10.1136/bmjopen-2024-087589)
Supplement: online supplemental file 1 [file bmjopen-15-3-s001.pdf]

Supplemental material

Table of contents

NAM analysis; characteristics of the 15-25 cohort (Table S1)……………………………………………………………2

Probabilities for falling within NAM recommendations by BMI for GWG between 15-25 weeks (Table S2) …….……3

Inter-rater agreement between 10-15 and 15-20 weeks GWG (Table S3)..4

Inter-rater agreement between gestation weeks >37 and ≥39 (Table S4)..………………………………………………...5

Maternal characteristics associated with NAM classifications for gestational age 15-20 weeks (FigureS1)…………....6

Maternal characteristics associated with NAM classifications for gestational age 10-15 weeks (Figure S2)…………...7

ROC curve using GWG 15-25 weeks (Figure S3)…..……….. ……………………………………. ………………………8

ROC curve using GWG 15-20 weeks (Figure S4) …...…………………………………………………………………….9

ROC curve using GWG 10-15 weeks (FigureS5).…………………….……………………………………………….....10

Number of visits by Socioeconomic status (Figure S6).……………..………………………………………………………11

Internal validation R code………………………………………………………….…………………………………12

Abbreviations

GWG - Gestational Weight Gain

BMI - Body Mass Index

ROC/AUC – Receiver Operating Characteristic Curve/ Area Under the Curve

NAM/IOM- National Academy of Medicine/ Institute of Medicine

Table S1. NAM analysis; characteristics of the 15-25 cohort

Characteristic	GWG Below NAM (n=3745, 32.2%)	GWG Within NAM (n= 4021, 34.5%)	GWG Exceed NAM (n=3873, 33.3%)	P value
**Age (years)**				
<25	522 (13.9)	500 (12.4)	626 (16.2)	
25-29	1,062 (28.4)	1,208 (30.0)	1,288 (33.3)	<0.001[a]
30-34	1,343 (35.9)	1,527 (38.0)	1,317 (34.0)	
≥35	818 (21.8)	786 (19.6)	642 (16.6)	
**BMI categories**				
Underweight (<18.5)	2,361(63.0)	2,154 (53.6)	1,214 (31.4)	
Normal weight (18.5 to 25.9)	146 (3.9)	136(3.4)	42 (1.1)	
Overweight (26.0 to 29.9)	681 (18.2)	1,118 (27.8)	1,563 (40.4)	<0.001[a]
Obese (≥30)	557 (14.9)	613 (15.2)	1,054 (27.2)	
**Offspring birth weight (g) (mean, SD)**	3272 (441)	3408 (432)	3540 (457)	<0.001[b]
**Parity (%)**				
0	1,424 (38.0)	1,702 (42.3)	1,912 (49.4)	
1-3	2,156 (57.6)	2,180 (54.2)	1,864 (48.1)	<0.001[a]
>3	165 (4.4)	139 (3.5)	97 (2.5)	
**Mothers weight (mean, SD)**	65.6 (17.4)	66.3 (15.3)	73.4 (16.8)	<0.001[b]
**COB (%)**				
Australian/European	1,183 (31.6)	1,375 (34.2)	1,375 (34.2)	
Polynesian	53 (1.4)	46 (1.1)	46 (1.1)	
South-East Asian	436 (11.6)	425 (10.6)	425 (10.6)	
Maritime SE Asian	199 (5.3)	187 (4.7)	187 (4.7)	
NE Asian	191 (5.1)	211 (5.3)	211 (5.3)	
SC. Asian	913 (24.4)	967 (24.1)	967 (24.1)	
African	182 (4.9)	147 (3.7)	147 (3.7)	<0.001[a]
Indigenous/Torres Strait Isle	28 (0.8)	20 (0.5)	20 (0.5)	
South Central America	22 (0.6)	23 (0.6)	23 (0.6)	
Central Asian	373 (10.0)	399 (9.9)	399 (9.9)	
South-eastern Europe	95 (2.5)	134 (3.3)	134 (3.3)	
North Africa and Mid. East	33 (0.9)	60 (1.5)	60 (1.5)	
Other [c]	37 (1.0)	27 (0.7)	27 (0.7)	
**Socio economic deprivation quintile (1=high, 5=low)**				
1	1,062 (28.4)	1,087 (27.0)	965 (24.9)	
2	233 (6.2)	255 (6.3)	211 (5.5)	
3	1,399 (37.4)	1,492 (37.1)	1,470 (38.0)	<0.001[a]
4	288 (7.7)	348 (8.7)	338 (8.7)	
5	763 (20.4)	839 (20.9)	889 (23.0)	
**No. of visits for the duration of pregnancy**				
1-3	1,321 (35.3)	981 (24.4)	881 (22.8)	
4-7	581 (15.5)	651 (16.2)	632 (16.3)	<0.001[a]
≥ 8	1,843 (49.2)	2,389 (59.4)	2,360 (60.9)	
**Maximum GWG(Kg) 10-15 weeks n (mean; 95%CI)**	8.5(8.0 to 9.0)	12.4 (1.9 to 12.8)	17.0 (16.4 to 17.6)	<0.001[b]
**Maximum GWG(Kg) 15-20 weeks n (mean; 95%CI)**	7.9 (7.4 to 8.4)	11.7 (11.8 to 12.1)	15.9 (15.3 to 16.6)	<0.001[b]
**Maximum GWG(Kg) 15-25 weeks n (mean; 95%CI)**	6.9 (6.4 to 7.3)	10.4 (9.9 to 10.9)	15.7 (14.8 to 16.6)	<0.001[b]

a. t-test was used to determine the *P* value,
b. Analysis of variance was used to determine the *P* value.
c. Other include Bahamas, Brunei, Darussalam, Dominican Republic, East Timor, Equatorial Guinea, Israel, Lesotho, Malta, South Africa and Not stated.

Table S2. Probabilities for falling within NAM recommendations by BMI for GWG between 15-25 weeks.

GWG between 15-25 weeks	Underweight			Normal weight			Overweight			Obese		
**Kg**	**Probability**	**95% CI**		**Probability**	**95% CI**		**Probability**	**95% CI**		**Probability**	**95% CI**	
**-20**	0.001	0.000	0.001	0.00	0.00	0.00	0.018	0.013	0.022	0.019	0.004	0.033
**-18**	0.001	-0.001	0.002	0.00	0.00	0.00	0.025	0.019	0.031	0.028	0.008	0.048
**-16**	0.002	-0.001	0.004	0.01	0.00	0.01	0.036	0.028	0.044	0.042	0.016	0.069
**-14**	0.003	-0.001	0.007	0.01	0.01	0.01	0.052	0.042	0.062	0.063	0.029	0.097
**-12**	0.006	-0.001	0.013	0.02	0.01	0.02	0.073	0.061	0.085	0.093	0.051	0.134
**-10**	0.011	0.000	0.022	0.03	0.02	0.03	0.102	0.088	0.116	0.133	0.085	0.182
**-8**	0.020	0.003	0.038	0.05	0.04	0.05	0.140	0.125	0.156	0.186	0.135	0.237
**-6**	0.037	0.012	0.063	0.08	0.07	0.09	0.188	0.172	0.204	0.249	0.201	0.297
**-4**	0.067	0.031	0.104	0.12	0.11	0.14	0.244	0.229	0.259	0.311	0.272	0.350
**-2**	0.118	0.072	0.164	0.19	0.18	0.21	0.304	0.291	0.316	0.357	0.329	0.384
**0**	0.198	0.148	0.247	0.28	0.27	0.30	0.358	0.349	0.367	0.369	0.350	0.389
**2**	0.306	0.263	0.349	0.38	0.37	0.39	0.398	0.391	0.405	0.344	0.328	0.361
**4**	0.425	0.387	0.463	0.46	0.45	0.47	0.415	0.408	0.423	0.293	0.273	0.313
**6**	0.517	0.469	0.564	0.49	0.48	0.50	0.409	0.399	0.418	0.231	0.206	0.256
**8**	0.546	0.481	0.612	0.46	0.44	0.48	0.381	0.368	0.395	0.173	0.145	0.200
**10**	0.511	0.407	0.614	0.39	0.37	0.42	0.341	0.324	0.358	0.124	0.097	0.152
**12**	0.432	0.283	0.581	0.31	0.28	0.34	0.294	0.273	0.314	0.087	0.063	0.112
**14**	0.338	0.158	0.518	0.23	0.19	0.26	0.246	0.224	0.269	0.061	0.040	0.081
**16**	0.249	0.063	0.436	0.16	0.13	0.19	0.202	0.179	0.225	0.041	0.025	0.058
**18**	0.176	0.004	0.348	0.11	0.08	0.14	0.163	0.140	0.186	0.028	0.015	0.041
**20**	0.121	-0.025	0.266	0.07	0.05	0.10	0.130	0.108	0.152	0.019	0.009	0.029
**22**	0.081	-0.035	0.196	0.05	0.03	0.07	0.103	0.083	0.122	0.013	0.005	0.020
**24**	0.053	-0.035	0.141	0.03	0.02	0.04	0.080	0.063	0.098	0.009	0.003	0.014
**26**	0.035	-0.030	0.100	0.02	0.01	0.03	0.063	0.047	0.078	0.006	0.002	0.010
**28**	0.022	-0.024	0.069	0.01	0.01	0.02	0.049	0.036	0.062	0.004	0.001	0.007
**30**	0.015	-0.019	0.048	0.01	0.00	0.01	0.038	0.026	0.049	0.003	0.000	0.005
**32**	0.009	-0.014	0.032	0.01	0.00	0.01	0.029	0.020	0.038	0.002	0.000	0.003
**34**	0.006	-0.010	0.022	0.00	0.00	0.01	0.022	0.015	0.030	0.001	0.000	0.002

Table S3 Inter-rater agreement between 10-15 and 15-20 weeks GWG.

Agreement	Expected agreement	Kappa	Std. Err.	Z	Prob>Z
74.87%	47.01%	0.5258	0.0419	12.56	0

Table S4 Inter-rater agreement between gestation weeks >37 and ≥39 (10-15 weeks).

Agreement	Expected Agreement	Kappa	Std. Err.	Z	Prob>Z
95.52%	47.19%	0.9151	0.007	130.73	0

Figure S1 Maternal characteristics associated with NAM classifications for gestational age 15-20 weeks.

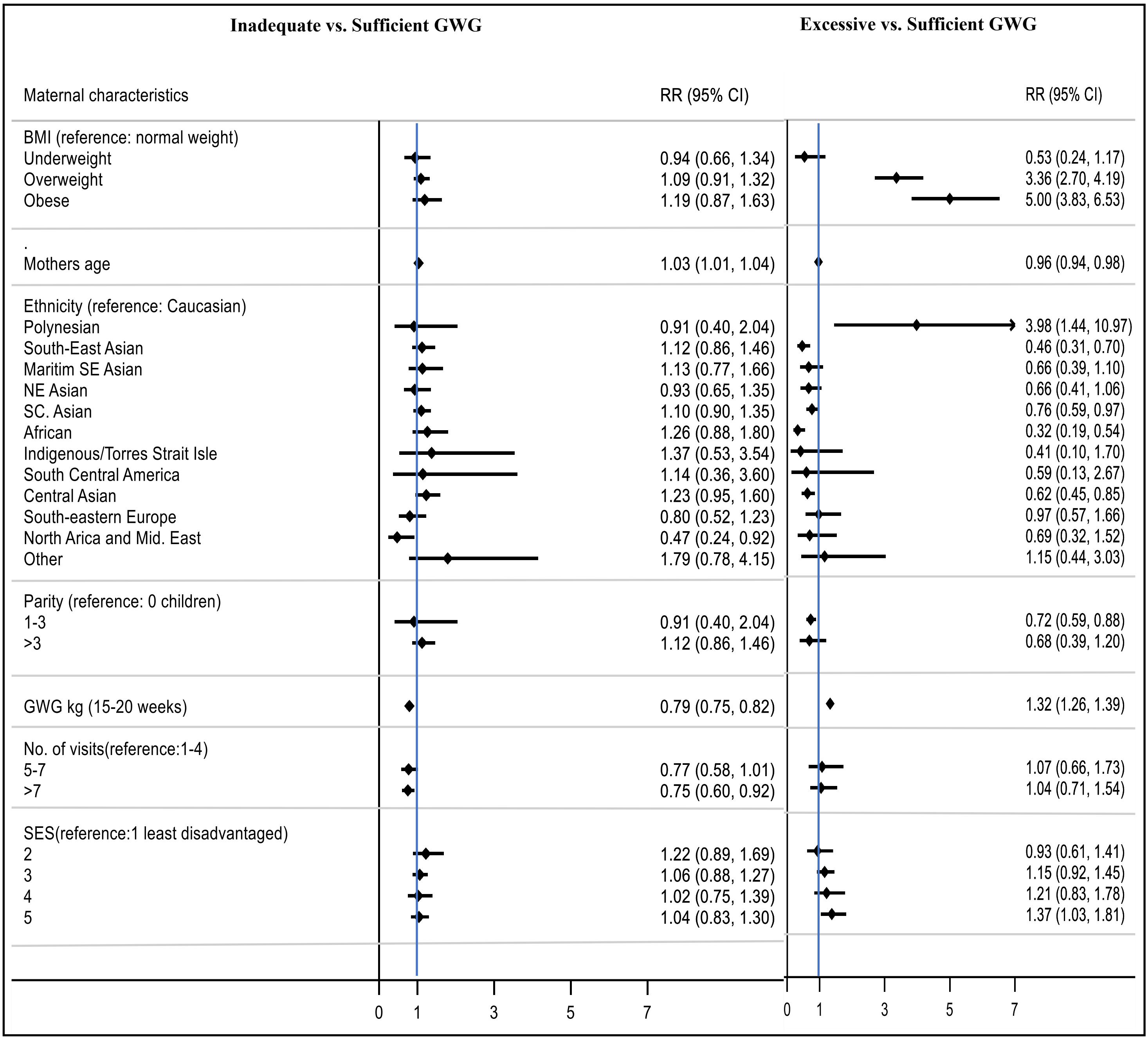

Figure S2 Maternal characteristics associated with NAM classifications for gestational age 10-15 weeks.

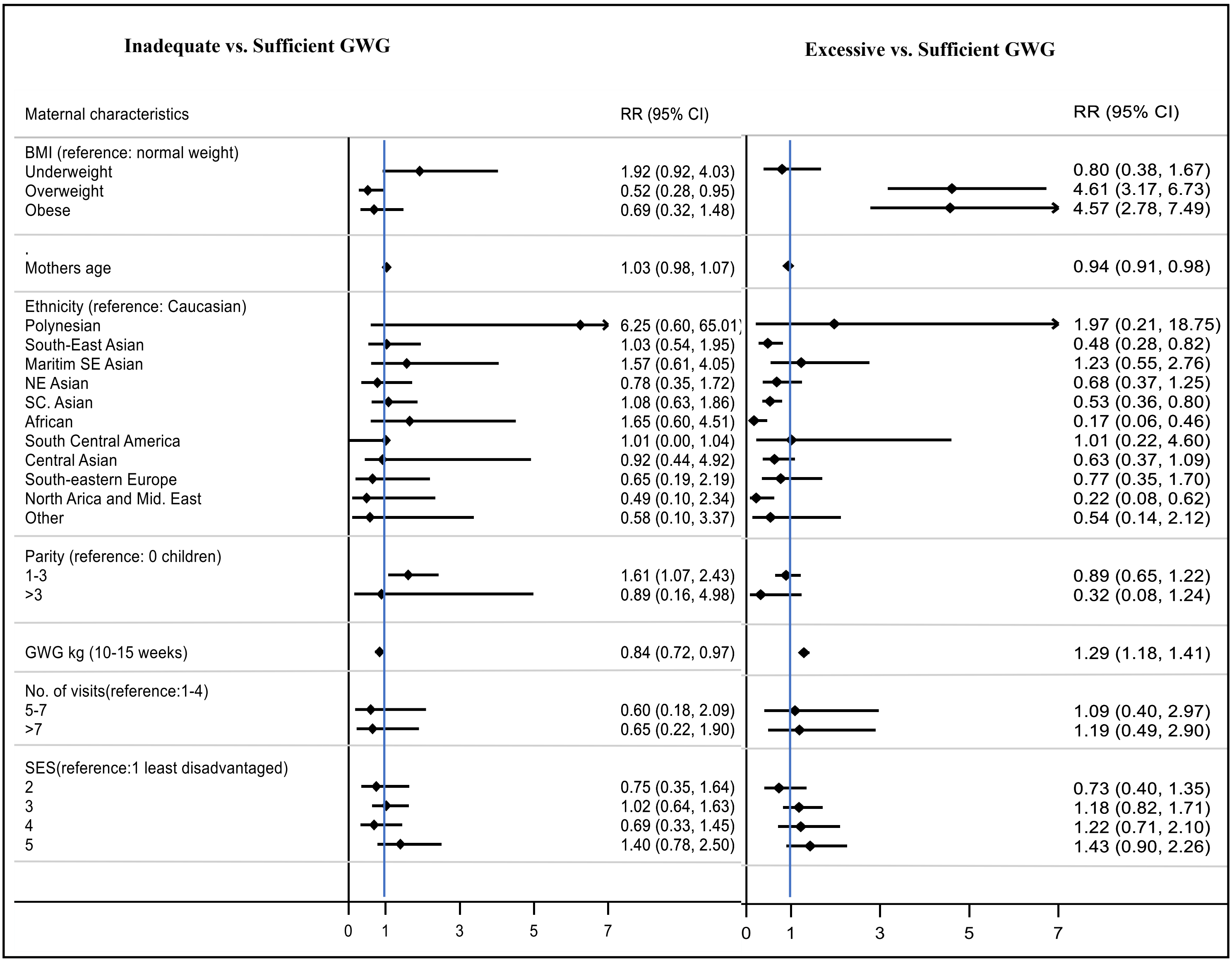

Figure S3 ROC curve using GWG 15-25 weeks

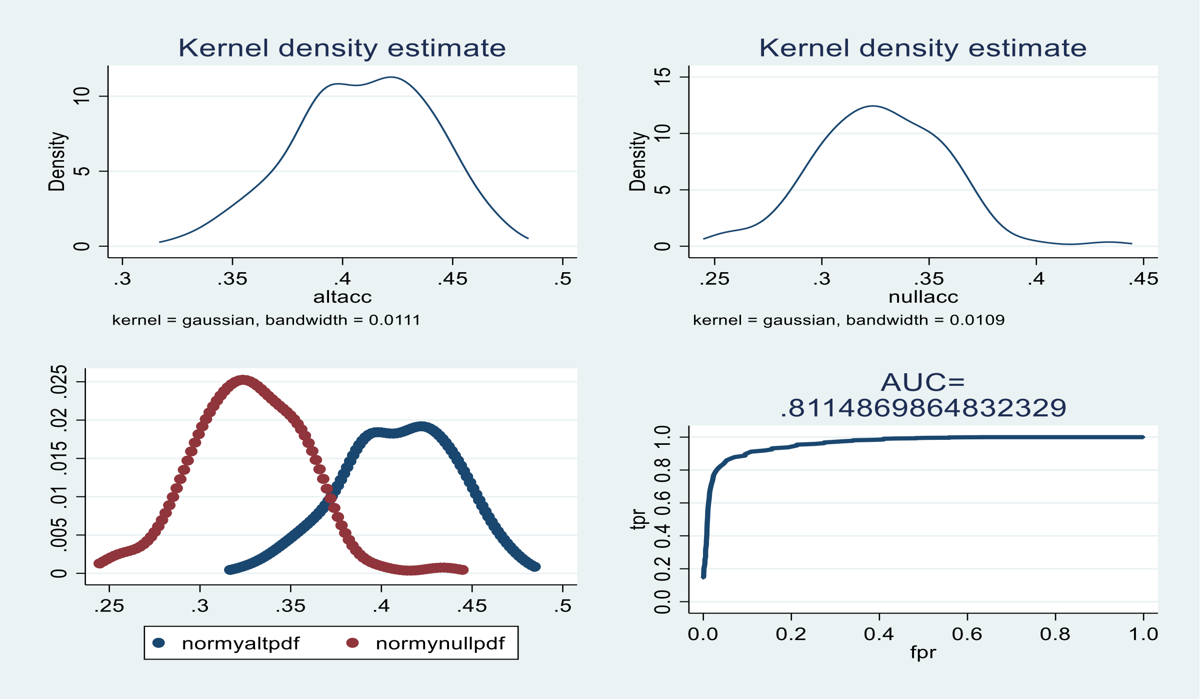

Figure S4 ROC curve using GWG 15-20 weeks

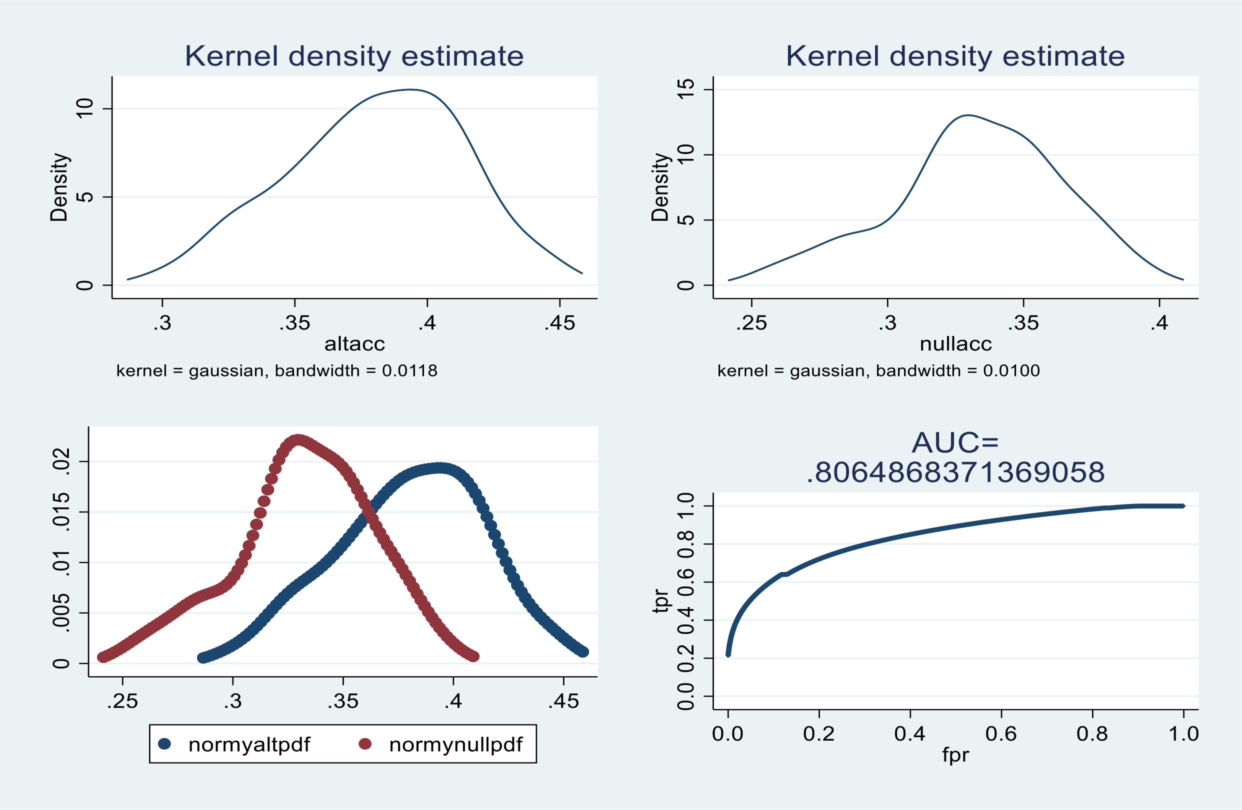

Figure S5 ROC curve using GWG 10-15 weeks

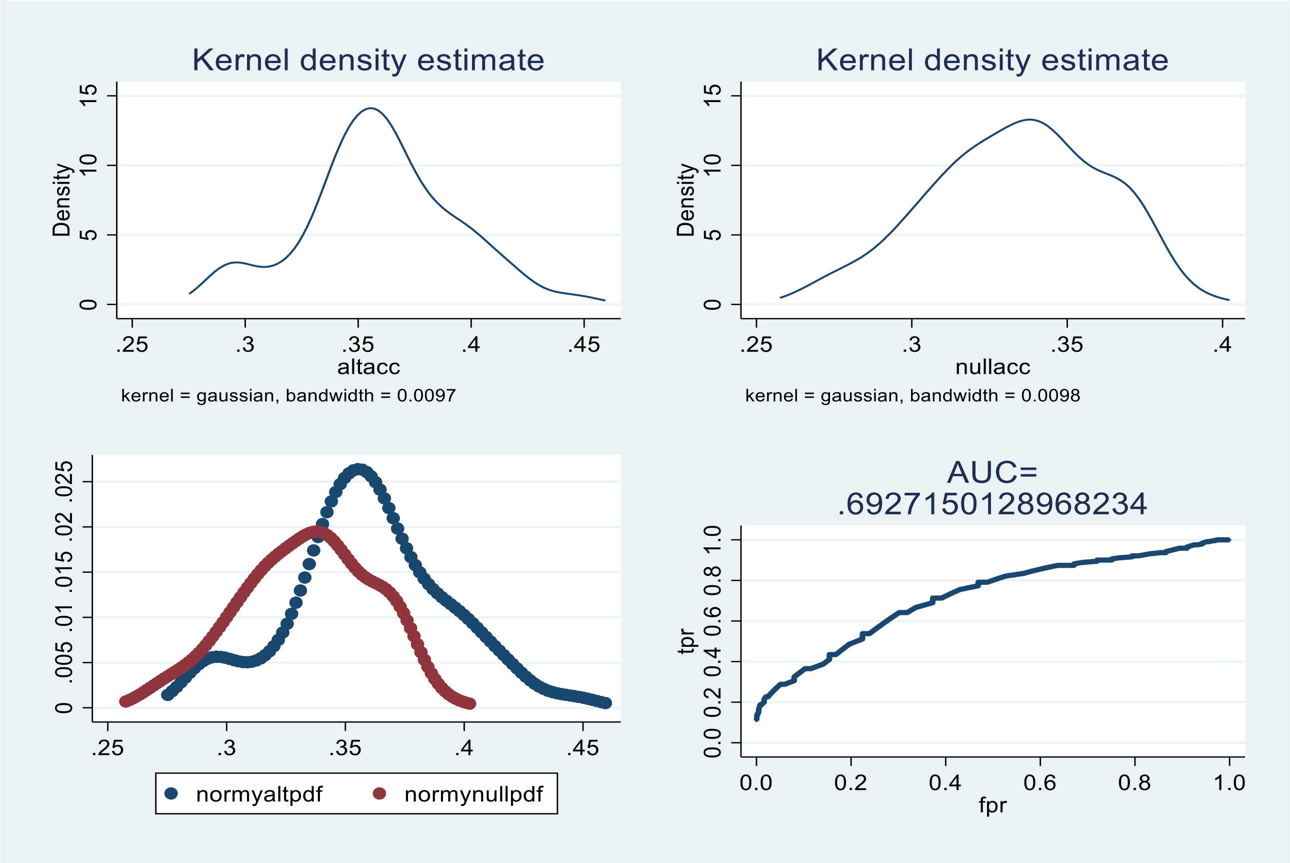

Figure S6 Number of visits (1-4, 5-7, >7) by Socioeconomic status (1 highest - 5 lowest).

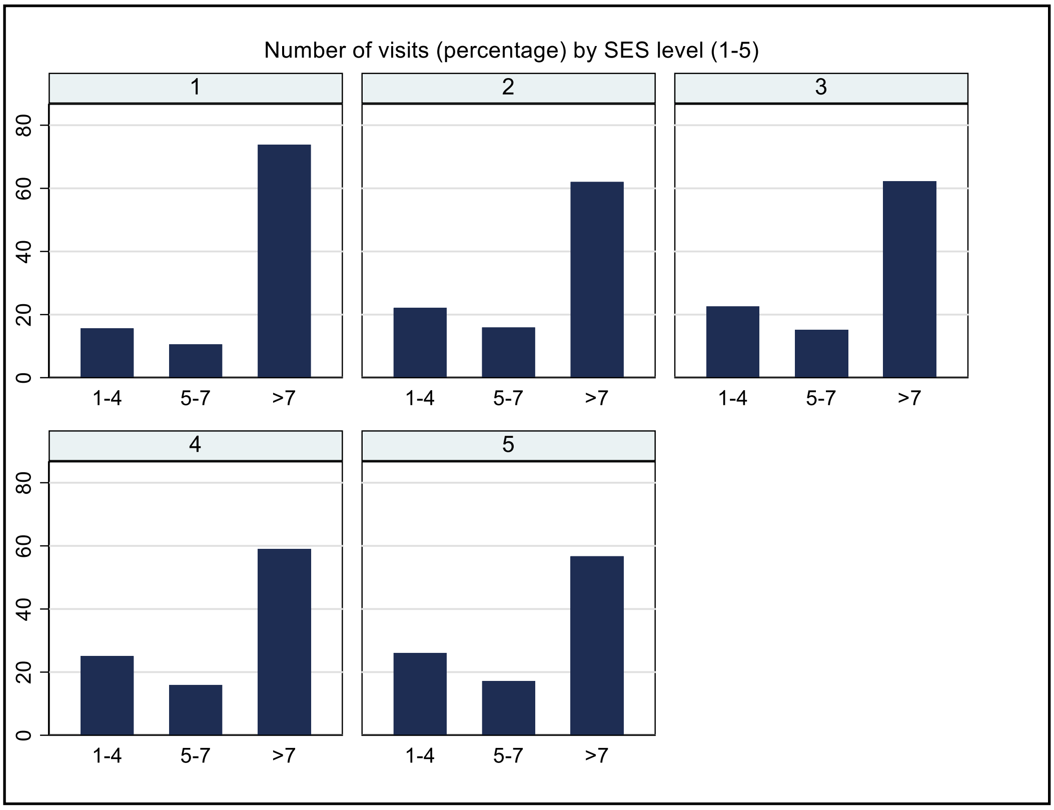

Internal validation

15–25-week gestational age cohort

```
#####################################################################################
> train <- sample_frac(multi, 0.60)
> sample_id <- as.numeric(rownames(train))
> test <- multi[-sample_id,]
> # Setting the basline
> train$iom1 <- relevel(train$iom1, ref = "within")
>
>
> multinom.fit <- multinom(iom1~ bmi_multi+ mother_age +ethcat2+ parity_new+gwg_15_25 , data = trai
n)
# weights:  63 (40 variable)
initial  value 4798.738477
iter  10 value 3977.678168
iter  20 value 3828.329869
iter  30 value 3816.004714
iter  40 value 3815.628865
final  value 3815.627721
converged
>
> # Checking the model
> summary(multinom.fit)
Call:
multinom(formula = iom1 ~ bmi_multi + mother_age + ethcat2 +
    parity_new + gwg_15_25, data = train)

Coefficients:
             (Intercept) bmi_multi2 bmi_multi3 bmi_multi4  mother_age    ethcat22    ethcat23
insufficient  0.19526351 -0.4526733   1.298189   1.236580 -0.02457691 -0.2172922 -0.01360124
exceed       -0.03437195 -1.4571491   2.897948   3.141707 -0.06208369  0.4659424 -0.90727623
                ethcat24    ethcat25    ethcat26   ethcat27   ethcat28  ethcat29  ethcat210
insufficient  0.04591674  0.02664233 -0.01908873 -0.3196837 -0.2224802 0.9714392 -0.2970821
exceed       -0.19016755 -0.05315818 -0.41721675 -0.8224812 -1.1951781 0.3642390 -0.6838491
             ethcat211 ethcat212   ethcat213 parity_new2 parity_new3 gwg_15_25
insufficient 0.6856019 1.0005385 -0.66741828  -0.3458577   -0.691610 0.2856105
exceed       0.5333558 0.5244279  0.07152858  -0.8330186   -1.326211 0.5435595

Std. Errors:
             (Intercept) bmi_multi2 bmi_multi3 bmi_multi4  mother_age  ethcat22  ethcat23
```

```
insufficient   0.2635528  0.1999643  0.1089659  0.1348872 0.009161691 0.4321756 0.1397381
exceed         0.3046259  0.3289172  0.1270853  0.1510052 0.010675195 0.4146780 0.1923819
              ethcat24   ethcat25   ethcat26   ethcat27   ethcat28   ethcat29 ethcat210 ethcat211
insufficient 0.2136962 0.2004129 0.1102201 0.2143670 0.5737458 0.7378618 0.1438448 0.2480397
exceed       0.2576938 0.2377412 0.1274293 0.2691439 0.7703845 0.9179972 0.1663343 0.2814803
             ethcat212 ethcat213 parity_new2 parity_new3  gwg_15_25
insufficient 0.4020904 0.4589302  0.09075488   0.2427314 0.01743003
exceed       0.4811605 0.4531095  0.10578305   0.2845319 0.02038242

Residual Deviance: 7631.255
AIC: 7711.255
>
>
>
> ## extracting coefficients from the model and exponentiate
> exp(coef(multinom.fit))
             (Intercept) bmi_multi2 bmi_multi3 bmi_multi4 mother_age  ethcat22  ethcat23
insufficient   1.2156313  0.6359258   3.662657   3.443815  0.9757226 0.8046948 0.9864908
exceed         0.9662121  0.2328993  18.136897  23.143348  0.9398042 1.5935153 0.4036221
              ethcat24 ethcat25  ethcat26  ethcat27  ethcat28 ethcat29 ethcat210 ethcat211
insufficient 1.0469872  1.02700 0.9810923 0.7263787 0.8005309 2.641744 0.7429830  1.984966
exceed       0.8268206  0.94823 0.6588781 0.4393402 0.3026500 1.439418 0.5046707  1.704643
             ethcat212 ethcat213 parity_new2 parity_new3 gwg_15_25
insufficient  2.719746 0.5130314   0.7076131   0.5007692  1.330574
exceed        1.689492 1.0741489   0.4347350   0.2654813  1.722126
>
>
> head(probability.table <- fitted(multinom.fit))
       within insufficient     exceed
1  0.09979072    0.2555404 0.64466893
6  0.17977437    0.5689116 0.25131407
7  0.17122449    0.3763405 0.45243505
9  0.77729559    0.2050750 0.01762941
11 0.07883099    0.1584980 0.76267096
13 0.69719218    0.2746490 0.02815879
>
> # Predicting the values for train dataset
> train$precticed <- predict(multinom.fit, newdata = train, "class")
>
> # Building classification table
> ctable <- table(train$iom1, train$precticed)
>
> # Calculating accuracy - sum of diagonal elements divided by total obs
> round((sum(diag(ctable))/sum(ctable))*100,2)
[1] 57.39
>
> # Predicting the values for train dataset
> test$pr <- predict(multinom.fit, newdata=test, "class")
>
> # Building classification table
> ctable1 <- table(test$iom1, test$pr)
```

```
>
> # Calculating accuracy - sum of diagonal elements divided by total obs
> round((sum(diag(ctable1))/sum(ctable1))*100,2)
[1] 88.04
```

15–20-week gestational age cohort

```
> train <- sample_frac(multi, 0.60)
> sample_id <- as.numeric(rownames(train))
> test <- multi[-sample_id,]
> # Setting the basline
> train$iom1 <- relevel(train$iom1, ref = "within")
>
>
```

```
> multinom.fit <- multinom(iom1~ bmi_multi+ mother_age +ethcat2+ parity_new+gwg_15_20 , data = trai
n)
# weights:  63 (40 variable)
initial  value 3199.158985
iter  10 value 2778.376085
iter  20 value 2717.022372
iter  30 value 2707.040822
iter  40 value 2706.323868
final  value 2706.323716
converged
>
> # Checking the model
> summary(multinom.fit)
Call:
multinom(formula = iom1 ~ bmi_multi + mother_age + ethcat2 +
    parity_new + gwg_15_20, data = train)

Coefficients:
             (Intercept) bmi_multi2 bmi_multi3 bmi_multi4  mother_age  ethcat22   ethcat23
insufficient   0.9267092 -0.7675457   1.066881  0.6528803 -0.02942670 0.2587476 -0.2371054
exceed         1.3362704 -1.6433024   2.447815  2.3635727 -0.07304881 1.2124945 -1.1048888
               ethcat24   ethcat25   ethcat26   ethcat27   ethcat28   ethcat29   ethcat210
insufficient -0.1628234 0.07075194 -0.0426440 -0.4569259 -0.7000788 -0.7526820 -0.09834994
exceed       -0.3807861 0.06861091 -0.3169907 -1.3423105 -1.4327918 -0.7661244 -0.64946547
             ethcat211 ethcat212  ethcat213 parity_new2 parity_new3 gwg_15_20
insufficient 0.4897353 0.9142551 -0.9131365  -0.3839477  -0.5113614 0.2431461
exceed       0.5167868 0.6568640 -0.2216876  -0.7578412  -1.0572698 0.5208593

Std. Errors:
             (Intercept) bmi_multi2 bmi_multi3 bmi_multi4 mother_age  ethcat22  ethcat23
```

```
insufficient   0.3158524  0.2545331  0.1245128  0.1497529 0.01087694 0.5672026 0.1623578
exceed         0.3583610  0.4606839  0.1418031  0.1605630 0.01253647 0.5365288 0.2284010
                ethcat24   ethcat25   ethcat26   ethcat27   ethcat28   ethcat29  ethcat210 ethcat211
insufficient 0.2600003 0.2420954 0.1304123 0.2357937 0.5698682 0.7921948 0.1652823 0.2997074
exceed       0.3104062 0.2865137 0.1472902 0.3011632 0.6651673 0.8180351 0.1942986 0.3230695
             ethcat212 ethcat213 parity_new2 parity_new3  gwg_15_20
insufficient 0.4600722 0.5198024   0.1056709   0.2735772 0.02837087
exceed       0.5389282 0.4791274   0.1212623   0.3201061 0.03222348

Residual Deviance: 5412.647
AIC: 5492.647
>
>
>
> ## extracting coefficients from the model and exponentiate
> exp(coef(multinom.fit))
             (Intercept) bmi_multi2 bmi_multi3 bmi_multi4 mother_age ethcat22  ethcat23
insufficient    2.526182  0.4641508   2.906299   1.921066  0.9710021 1.295307 0.7889081
exceed          3.804827  0.1933405  11.563058  10.628858  0.9295555 3.361860 0.3312477
              ethcat24 ethcat25  ethcat26  ethcat27  ethcat28  ethcat29 ethcat210 ethcat211
insufficient 0.8497412 1.073315 0.9582525 0.6332272 0.4965462 0.4711014 0.9063317  1.631884
exceed       0.6833240 1.071019 0.7283375 0.2612414 0.2386417 0.4648110 0.5223249  1.676632
             ethcat212 ethcat213 parity_new2 parity_new3 gwg_15_20
insufficient  2.494916 0.4012637   0.6811671   0.5996786  1.275255
exceed        1.928734 0.8011656   0.4686771   0.3474030  1.683474
>
>
> head(probability.table <- fitted(multinom.fit))
       within insufficient    exceed
3  0.12611719    0.2607147 0.6131681
4  0.34668290    0.4173697 0.2359474
13 0.39449080    0.4148164 0.1906928
19 0.06266869    0.2698881 0.6674432
21 0.09180914    0.3268654 0.5813255
22 0.05650859    0.2631226 0.6803688
>
> # Predicting the values for train dataset
> train$precticed <- predict(multinom.fit, newdata = train, "class")
>
> # Building classification table
> ctable <- table(train$iom1, train$precticed)
>
> # Calculating accuracy - sum of diagonal elements divided by total obs
> round((sum(diag(ctable))/sum(ctable))*100,2)
[1] 54.05
>
> # Predicting the values for train dataset
> test$pr <- predict(multinom.fit, newdata=test, "class")
>
> # Building classification table
> ctable1 <- table(test$iom1, test$pr)
```

```
>
> # Calculating accuracy - sum of diagonal elements divided by total obs
> round((sum(diag(ctable1))/sum(ctable1))*100,2)
[1] 80.3
```

10–15-week gestational age cohort

```
> train <- sample_frac(multi, 0.60)
> sample_id <- as.numeric(rownames(train))
> test <- multi[-sample_id,]
> # Setting the basline
> train$iom1 <- relevel(train$iom1, ref = "within")
>
```

```
> multinom.fit <- multinom(iom1~ bmi_multi+ mother_age +ethcat2+ parity_new+gwg_10_15 , data = trai
n)
# weights:  63 (40 variable)
initial  value 1110.697024
iter  10 value 927.825997
iter  20 value 884.101136
iter  30 value 883.469877
iter  40 value 883.296333
iter  50 value 883.285179
final  value 883.285138
converged
>
> # Checking the model
> summary(multinom.fit)
Call:
multinom(formula = iom1 ~ bmi_multi + mother_age + ethcat2 +
    parity_new + gwg_10_15, data = train)

Coefficients:
             (Intercept) bmi_multi2 bmi_multi3 bmi_multi4  mother_age   ethcat22    ethcat23
insufficient    2.972227 -0.1527887  0.8529476   0.394777 -0.07345998 -1.5253658 -0.05195245
exceed          3.449504 -0.5373756  2.4485759   2.144314 -0.10947635 -0.3654632 -0.83189325
               ethcat24  ethcat25   ethcat26   ethcat27  ethcat28 ethcat29  ethcat210
insufficient -0.3073513 0.5309281 -0.2883625 -0.6362737 -5.876792 21.92112  0.1252975
exceed       -0.5629021 0.3124770 -0.7435594 -1.9770203  7.059258 22.46126 -0.4212757
              ethcat211  ethcat212 ethcat213 parity_new2 parity_new3  gwg_10_15
insufficient  0.1258363  0.5883913 0.7987576  -0.2007135  -0.0368313 0.07025592
```

```
exceed       -0.3068314 -0.4880200 0.5701357  -0.5596843  -1.8166484 0.39747802

Std. Errors:
             (Intercept) bmi_multi2 bmi_multi3 bmi_multi4 mother_age ethcat22  ethcat23
insufficient   0.7096218  0.3746096  0.2916545  0.3780974 0.02291762 1.252948 0.3003612
exceed         0.7445813  0.4481810  0.2935885  0.3613506 0.02441095 1.000435 0.3403366
              ethcat24  ethcat25  ethcat26  ethcat27     ethcat28  ethcat29 ethcat210 ethcat211
insufficient 0.4642054 0.4413926 0.2639223 0.5210615 4.546459e-09 0.4474272 0.3689490 0.5225026
exceed       0.5033568 0.4663588 0.2722367 0.6172359 1.367892e-06 0.4474272 0.3918299 0.5426384
             ethcat212 ethcat213 parity_new2 parity_new3  gwg_10_15
insufficient 0.8101594  1.150238   0.2074300   0.6429297 0.06966230
exceed       0.8812808  1.177546   0.2206491   0.8052081 0.06866262

Residual Deviance: 1766.57
AIC: 1846.57
>
>
>
> ## extracting coefficients from the model and exponentiate
> exp(coef(multinom.fit))
             (Intercept) bmi_multi2 bmi_multi3 bmi_multi4 mother_age  ethcat22  ethcat23
insufficient    19.53538  0.8583111   2.346553   1.484053  0.9291733 0.2175415 0.9493740
exceed          31.48477  0.5842796  11.571855   8.536187  0.8963034 0.6938751 0.4352245
              ethcat24 ethcat25  ethcat26  ethcat27     ethcat28   ethcat29 ethcat210 ethcat211
insufficient 0.7353922 1.700510 0.7494899 0.5292609 2.803766e-03 3313001665 1.1334856 1.1340965
exceed       0.5695537 1.366807 0.4754187 0.1384813 1.163581e+03 5685934823 0.6562091 0.7357746
             ethcat212 ethcat213 parity_new2 parity_new3 gwg_10_15
insufficient 1.8010888  2.222778   0.8181468   0.9638387  1.072783
exceed       0.6138406  1.768507   0.5713894   0.1625697  1.488067
>
>
> head(probability.table <- fitted(multinom.fit))
        within insufficient    exceed
1  0.070627951    0.2708116 0.6585604
6  0.209304351    0.2626310 0.5280647
8  0.012777300    0.1391523 0.8480704
25 0.005628915    0.0474674 0.9469037
32 0.211737134    0.4551236 0.3331393
40 0.026185720    0.2197397 0.7540746
>
> # Predicting the values for train dataset
> train$precticed <- predict(multinom.fit, newdata = train, "class")
>
> # Building classification table
> ctable <- table(train$iom1, train$precticed)
>
> # Calculating accuracy - sum of diagonal elements divided by total obs
> round((sum(diag(ctable))/sum(ctable))*100,2)
[1] 59.15
>
> # Predicting the values for train dataset
```

```
> test$pr <- predict(multinom.fit, newdata=test, "class")
>
> # Building classification table
> ctable1 <- table(test$iom1, test$pr)
>
> # Calculating accuracy - sum of diagonal elements divided by total obs
> round((sum(diag(ctable1))/sum(ctable1))*100,2)
[1] 61.43
```

Supplementary Material
