# Supplementary material for: Predictors of inadequate and excessive gestational weight gain in women: a retrospective longitudinal observational study
Source: BMJ Open. 2025 Mar 22;15(3):e087589. doi: 10.1136/bmjopen-2024-087589 (PMC11931920; doi:10.1136/bmjopen-2024-087589)
Supplement: Supplementary file 2 [file bmjopen-2024-087589.reviewer_comments.pdf]

## PEER REVIEW HISTORY

BMJ Open publishes all reviews undertaken for accepted manuscripts. Reviewers are asked to complete a checklist review form and are provided with free text boxes to elaborate on their assessment. These free text comments are reproduced below.

### ARTICLE DETAILS

**Title (Provisional)**

Predictors of inadequate and excessive gestational weight gain in women: a retrospective longitudinal observational study.

**Authors**

Ranasinha, Sanjeeva; Enticott, Joanne; Harrison, Cheryce; Teede, Helena J.

---

### VERSION 1 - REVIEW

---

| | |
|---|---|
| **Reviewer** | **1** |
| **Name** | **Gherasim, , Andreea** |
| **Affiliation** | **Grigore T Popa University of Medicine and Pharmacy Iasi Faculty of Medicine** |
| **Date** | **09-May-2024** |
| **COI** | **None** |

---

The article offers a detailed examination of the predictors of both inadequate and excessive gestational weight gain in women; this topic has a significant relevance. It commendably outlines the methodology, statistical analysis, results, conclusions and the study's strenghts and limitations. While the references cited are pertinent to the research, some are dated and do not include the most recent decade's literature. The references could be updated. Overall, I believe the article is well-done.

---

| | |
|---|---|
| **Reviewer** | **2** |
| **Name** | **Asefa, Fekede** |
| **Affiliation** | **Haramaya University, School of Public Health** |
| **Date** | **20-Jul-2024** |
| **COI** | **I have no conflict of interest to declare.** |

---

Thank you for the opportunity to review this interesting manuscript by Ranasinha and colleagues. This study utilized routine health data from the Australian Monash Health Network birthing outcome system to explore risk factors associated with suboptimal

gestational weight gain (GWG) as recommended by the National Academy of Medicine (NAM). Specifically, it investigated GWG between 15-25 weeks of pregnancy and developed a GWG risk prediction tool using early GWG data at 10-15, 15-20, and 15-25 weeks. The study found a high prevalence of suboptimal GWG (64.3%). Additionally, it identified risk factors associated with both excessive and insufficient GWG, including pre-pregnancy BMI and maternal country of birth by region.

Strengths of this study include the use of a relatively large cohort of pregnant women from diverse backgrounds and the development of a risk prediction tool that demonstrated good performance and reliability. The manuscript is well-written, and the statistical methods are clearly described.

Please find my comments and suggestions below.

1. On page 3, line 50, you stated that 'The study included 17,397 women from 149 countries of diverse socioeconomic backgrounds...' to avoid confusion (readers may assume that the data were collected from 149 countries), you may specify that this diversity is based on country of birth (COB).

2. On page 5, line 12, you can provide a full description of "US."

3. Please ensure consistency in the formatting of numbers throughout the manuscript (e.g., use of a comma before three digits: 11,369 vs. 11369).

4. Please change the word 'Bivariate' to "Bivariable'.

5. The authors stated, "The multinomial model was used after detecting violations in the proportional odds assumption for an ordinal logistic regression." Given that using adequate GWG (which is not the lowest group) as a reference group is appropriate and logical, it is unclear why the authors initially preferred the ordinal logistic regression model.

6. Given that they used logistic regression, I suggest the authors ensure they actually calculated relative risk.

7. The authors identified that women of African origin had a higher risk of gaining weight below NAM recommendations compared to Caucasians. However, studies show that the majority of women from South Africa gain excess weight, unlike most other African countries. If data on the specific countries of origin are available and the sample size permits, I suggest the authors examine whether their findings also apply to women from South Africa. Furthermore, although most women from low and lower-middle-income African countries gain insufficient weight, evidence suggests that a majority of immigrant women from these countries in high-income settings gain excess weight. The authors may want to triangulate their findings from multiple perspectives.

8. On page 8, the sentence on lines 48-50 seems unclear. The authors may want to revise it.

9. The authors included GWG at 10-15 weeks, 20-25 weeks, and 15-25 weeks in the multinomial model as risk factors for inadequate and excess GWG. I find it difficult to understand the logic behind considering "GWG as a risk factor for GWG."

10. One of the age categories for the participants was "<25 years." What is the lowest age value? Are there any participants below 20 years? If that is the case, I wonder whether it might be preferable to use the WHO child growth standards (https://www.who.int/tools/growth-reference-data-for-5to19-years) to define BMI categories for this subgroup instead of using the fixed cutoff points for adults.

---

## VERSION 1 - AUTHOR RESPONSE

| **BMJ's research committee meeting: Comments** | Author response to the comments | Additional information |
|---|---|---|
| **Reviewer 1 (Dr. Andreea Gherasim)**<br>The article offers a detailed examination of the predictors of both inadequate and excessive gestational weight gain in women; this topic has a significant relevance. It commendably outlines the methodology, statistical analysis, results, conclusions and the study's strenghts and limitations. While the references cited are pertinent to the research, some are dated and do not include the most recent decade's literature. The references could be updated. Overall, I believe the article is well-done. | We conducted a search to find the most recent articles related to this study, and now included them as references in the manuscript. | 8. Bodnar LM, Johansson K, Himes KP, et al. Gestational weight gain below recommendations and adverse maternal and child health outcomes for pregnancies with overweight or obesity: a United States cohort study. The American Journal of Clinical Nutrition 2024; **120**(3): 638-47.<br>10. McDonald SD, Yu ZM, van Blyderveen S, et al. Prediction of excess pregnancy weight gain using psychological, physical, and social predictors: A validated model in a prospective cohort study. PLoS One 2020; **15**(6): e0233774. |
| **Reviewer 2 (Dr. Fekede Asefa)** | Thank you very much for your valuable comments and suggestions. | |
| 1. On page 3, line 50, you stated that 'The study included 17,397 women from 149 countries of diverse socioeconomic | Done | |

| | | |
|---|---|---|
| backgrounds…' to avoid confusion (readers may assume that the data were collected from 149 countries), you may specify that this diversity is based on country of birth (COB). | | |
| 2. On page 5, line 12, you can provide a full description of "US." | Done | |
| 3. Please ensure consistency in the formatting of numbers throughout the manuscript (e.g., use of a comma before three digits: 11,369 vs. 11369). | Done | |
| 4. Please change the word 'Bivariate' to "Bivariable'. | We changed the word bivariate to bivariable. | |
| 5. The authors stated, "The multinomial model was used after detecting violations in the proportional odds assumption for an ordinal logistic regression." Given that using adequate GWG (which is not the lowest group) as a reference group is appropriate and logical, it is unclear why the authors initially preferred the ordinal logistic regression model. | Good point. If possible, we prefer to leave this in the manuscript as it supports the approach of the multinomial model. | |
| 6. Given that they used logistic regression, I suggest the authors ensure they actually calculated relative risk. | Since we used multinomial logistic regression and not logistic regression, it is valid to express the output as Relative Risk Ratio (RRR). To enhance clarity, we replaced "RR" with "RRR."<br><br>The multinomial logit model estimates k-1 models, where the kth equation is relative to the referent group. The RR of a coefficient indicates how the risk of the outcome falling in the comparison group compared to the risk of the outcome falling | Reference: https://www.stata.com/products/stb/journals/stb53.pdf |

| | | |
|---|---|---|
| | in the referent group changes with the variable in question. | |
| 7. The authors identified that women of African origin had a higher risk of gaining weight below NAM recommendations compared to Caucasians. However, studies show that the majority of women from South Africa gain excess weight, unlike most other African countries. If data on the specific countries of origin are available and the sample size permits, I suggest the authors examine whether their findings also apply to women from South Africa. Furthermore, although most women from low and lower-middle-income African countries gain insufficient weight, evidence suggests that a majority of immigrant women from these countries in high-income settings gain excess weight. The authors may want to triangulate their findings from multiple perspectives. | Ancestry of the South African-born community in Australia is as follows: English 23.9%, Dutch 6.7%, Scottish 6.2%, other ancestry 31.2%, and South African 32.0%. Therefore, it is difficult to determine exactly the ethnicity as 32% who classify themselves as South African [1].<br><br>This is a limitation of our routinely collected data, and is now noted in the:<br><br>- STRENGTHS AND LIMITATIONS OF THIS STUDY box (third bullet point: • Classifying ethnicity by the country of birth may have introduced some inaccuracies therefore we aim to improve on this by exploring self-identified ethnicity in future.<br>- discussion section in the limitation paragraph. | [1] https://www.homeaffairs.gov.au/mca/files/2016-cis-south-africa.PDF |
| 8. On page 8, the sentence on lines 48-50 seems unclear. The authors may want to revise it. | Changed the sentence to improve clarity. | |
| 9. The authors included GWG at 10-15 weeks, 20-25 weeks, and 15-25 weeks in the multinomial model as risk factors for inadequate and excess GWG. I find it difficult to understand the logic behind considering | Good point. The logic is as follows:<br><br>- For an individual, her final GWG can be predicted using her weight gained earlier in the pregnancy.<br>- We can re-run the model throughout her pregnancy, using her most recent weight data. | |

| | | |
|---|---|---|
| "GWG as a risk factor for GWG." | <ul><li>We can run model as early as 15 weeks (inputting the greatest weight gain between 10-15wks).</li><li>At 20 weeks, model predictive performance improves (inputting the greatest weight gain between 15-20wks).</li><li>At 25 weeks, model predictive performance improves further (inputting the greatest weight gain between 15-25wks).</li></ul>This variable serves as an important predictor of both inadequate and excessive GWG in the model. For clarity, we have added this sentence into the results section under the heading, "Aim 2 Risk prediction algorithm": For an individual, her final GWG can be predicted using her weight gained earlier in the pregnancy. We examined the predictive performance using three times in early pregnancy. | |
| 10. One of the age categories for the participants was "<25 years." What is the lowest age value? Are there any participants below 20 years? If that is the case, I wonder whether it might be preferable to use the WHO child growth standards (https://www.who.int/tools/growth-reference-data-for-5to19-years) to define BMI categories for this subgroup instead of using the fixed cutoff points for adults. | Only 217 women, or 1.2%, are under 20 years old, and this has been added to Table 1. This low number makes it insufficient for a robust analysis. | |
